# The Survival and Physiological Response of *Calliptamus abbreviatus* Ikonn (Orthoptera: Acrididae) to Flavonoids Rutin and Quercetin

**DOI:** 10.3390/insects15020095

**Published:** 2024-02-01

**Authors:** Xunbing Huang, Li Zheng, Yueyue Wang

**Affiliations:** 1 College of Agriculture and Forestry Science, Linyi University, Linyi 276000, China; wangyueyue@lyu.edu.cn; 2 Key Laboratory of Natural Enemies Insects, Ministry of Agriculture and Rural Affairs, Jinan 250100, China

**Keywords:** plant-derived compounds, grasshopper control, survival, enzyme activities

## Abstract

**Simple Summary:**

It is of great significance to develop and utilize plant-derived compounds for the sustainable control of grasshopper. This study assessed the adverse effects of rutin and quercetin on grasshopper, as well as the insect’s physiological response to these two plant-derived compounds. Rutin and quercetin all exhibited toxic effects on grasshopper, with quercetin showing a stronger toxicity, which indicated that they—especially quercetin—have the potential to be developed as biopesticides to control the grasshopper.

**Abstract:**

Insect-resistant substances from plants are important natural resources that human beings can potentially develop and use to control pests. In this study, we explored the adverse effects of rutin and quercetin on grasshopper (*Calliptamus abbreviatus*), as well as the insect’s physiological response to these substances in laboratory and field experiments. These two plant compounds exhibited toxic effects on *C. abbreviatus*, with quercetin showing a stronger toxicity, indicated by a lower survival, slower development, and higher induced gene expression and activities of UDP-glucuronosyltransferase, cytochrome P450s, superoxide dismutase, peroxidase and catalase, compared to rutin. These compounds, especially quercetin, have the potential to be developed as biopesticides to control grasshoppers.

## 1. Introduction

*Calliptamus abbreviatus* (Orthoptera: Acrididae) is a dominant grasshopper species in grasslands in northern China. Its high occurrence negatively affects agricultural and animal husbandry production [1,2,3]. At present, the use of chemical pesticides is the main method for the control of *C. abbreviatus*. Although chemical pesticides play an important role in plant protection, their irrational use often leads to problems of pesticide residues, pest resistance and resurgence, which in turn adversely affects the ecosystem [4,5,6]. In recent decades, research efforts have focused on the identification of bio-pesticides that are low-toxicity, biodegradable and low-residue [7,8]. Insect-resistant substances from plants, such as flavonoids, terpenoids, alkaloids, and sterols, exhibit the above characteristics and are important natural resources to be developed as biological insecticides [9,10]. Therefore, it is of great significance to develop and utilize plant-derived compounds for the sustainable control of *C. abbreviatus*.

To resist insect feeding or other stress, plants produce a large number of compounds, serving as “chemical weapons”, to resist damage [11,12]. Some of these compounds can adversely affect the feeding behavior, growth, development or reproduction of insects, or even directly poison them [13]. For example, plant derived substances such as tannin, nicotine and matrine can directly hinder the normal growth and development of pests such as *Helicoverpa armigera*, *Papilio polyxenes*, *Bemisia tabaci*, *Spodoptera eridania* and *Pectinophora gossypiella* [14,15,16]. In addition, other compounds can be induced for the indirect control of pests by attracting predatory or parasitic natural enemies, such as that exhibited by some terpenoids on *Pyrausta nubilalis* and *Spodoptera exigua* [17,18]. The mechanism underlying the toxicity of plant secondary compounds on insects includes the inhibition of the activities of acetylcholinesterase, acetylcholine receptor, and enzyme activities; or destroying the aminobutyric acid gated chlorine channels, blocking Na^+^/K^+^ exchange and Ca^2+^ channels, and interfering with the series of reactions of respiration and tyramine and the balance of ecdysone or juvenile hormone [19]. Compounds such as azadirachtin can induce reactive oxygen species (ROS) in insects, resulting in oxidative damage, which inhibits the normal development of insects [20,21,22]. 

Herbivorous insects have evolved mechanisms to detoxify these toxic plant compounds to reduce their harm. These include the synthesis and secretion of cytochrome P450s, UDP-glucuronosyltransferase, glutathione sulfotransferase, carboxylesterase and other detoxification enzymes in guts and fat bodies [3,16,23,24,25]. Moreover, insects can also secrete peroxidase, catalase, superoxide dismutase and other protective enzymes to alleviate oxidative damage [26,27]. The changes in ROS content, gene expression or the activities of detoxification enzymes and protective enzymes in insects can indicate an adaptation response to the toxicities of these plant compounds. 

Rutin and quercetin are flavonoids which have been identified in a wide plant species. They can inhibit the normal growth and development of pests such as *Spodoptera litura*, *Lymantria dispar*, *Ostrinia nubilalis*, *Helicoverpa zea*, *Coptotermes formosanus*, *Pectinophora gossypiella* and *Acheta domesticus*, resulting in increased pest mortality, decreased development rate and fecundity [28,29,30,31]. Their adverse effects on pests suggest that they are an important resource, which can be potentially developed and used to control pests. In this study, we assessed the adverse effects of rutin and quercetin on *C. abbreviatus*, as well as the insect’s physiological response to these two plant-derived compounds. The aim of this research was to provide a theoretical basis for the further development and utilization of rutin or quercetin to control grasshopper.

## 2. Materials and Methods

### 2.1. Field Grasshopper Collection

Third instar nymphs of *C. abbreviatus* were collected from an alfalfa field by a sweep net (40 cm diameter) in Yishui, Shandong Province, northeastern China (35.839° N, 118.296° E) in June 2021 and 2022. The vegetation in this alfalfa field was dominated by *Medicago sativa* L. Collected nymphs were maintained temporarily in a cage, and then used in the indoor trial in 2021 and field cage study in 2022.

### 2.2. Indoor Feeding Trial

A total of 1600 healthy third instar nymphs of *C. abbreviatus* were selected and starved for 24 h to conduct the indoor feeding trial in June 2021. They were randomly assigned to 80 mesh cages (size: 40 cm × 30 cm × 20 cm), with each cage containing 20 nymphs. A series of rutin and quercetin concentrations (0%, 0.001%, 0.01%, 0.1% and 1.0%) were prepared using dimethyl sulfoxide (DMSO). For each cage, 10 mL of the prepared solution was evenly applied to 50 g of fresh *M. sativa* using a 50 mL hand sprayer, allowed to dry, and then provided to *C. abbreviatus*. Each treatment included 8 replicates (cages). Grasshopper survival was inspected daily until all surviving individuals became adults. For each treatment, 8 adult individuals were randomly collected to weigh the body mass (mg) and immediately frozen in liquid nitrogen. These frozen samples were stored at −80 °C and used to measure gene expressions, rutin and quercetin contents, ROS level and enzyme activity (see below). Grasshopper survival rate (%) and development time (days) were calculated using the described method by Li et al. [32]. The survival rate (%) of grasshoppers was calculated by the number of surviving adulthoods/numbers of initial third instar individuals (*n* = 20). Development time (days) of *C. abbreviatus* from third instar to adult was calculated by the formula: $DT=\frac{\sum_{i=1}^{n}i*N_{i}}{N_{t}}$, where *i* is the number of days from third instar to adult; *N_i_* is the number of grasshopper individuals with the development time corresponding to that value of “*i*”; *N_t_* is the number of grasshopper *C. abbreviates* that successfully survived to adulthood.

### 2.3. Gene Expression 

The relative gene expressions of β-glucosidase, UDP-glucuronosyltransferase, cytochrome P450 6k1, superoxide dismutase, peroxidase and catalase were measured using a real time PCR. The sequences of these genes were acquired from our previous published transcriptome profiles of *C. abbreviatus* (SRA database: SRR10289990). Gene-specific primers were designed using the Primer 3 software (version 4.1.0, http://bioinfo.ut.ee/primer3-0.4.0/, accessed on 20 January 2024) and have been provided in Supplementary Table S1. The same method of real time PCR was used, as described in our published paper [3]. In brief, the total RNA from each grasshopper sample was extracted using TRIzol reagent (Invitrogen, San Diego, CA, USA). After RNA extraction, the AMV reverse transcriptase (Invitrogen, San Diego, CA, USA) was used to synthesize cDNA. Experiments were performed using SYBR^®^ green PCR mix (Hilden, Germany) according to the manufacturer’s instructions. Relative gene expressions were calculated using the 2^−ΔΔCT^ method, with β-actin as the reference gene. Expression values were adjusted by setting the expression of controls to 1 for each gene. The PCR runs for each gene of each treatment included eight biological replicates with three technical replicates.

### 2.4. Analysis of Rutin and Quercetin Content in Grasshopper 

The rutin and quercetin content in each grasshopper sample for each treatment were measured by high-performance liquid chromatography (HPLC), using the same method described in a previously published paper [33]. In brief, the flavonoids in each grasshopper sample were extracted by soaking in 70% ethanol. After ultrasonic extraction for 35 min, the solution was put in a water bath at 60 °C to evaporate the ethanol. Anhydrous methanol was added until it reached a volume of 50.00 mL. After centrifugation, supernatant was collected and filtrated through a 0.45 μm filter membrane. Ultrasonic degassing was carried out to obtain the flavonoids extract. Each reference substance was dissolved by anhydrous methanol and filtrated by a 0.45 μm filter membrane. Finally, rutin and quercetin were detected by chromatographic experiments conducted using the Agilent 1260 (Santa Clara, CA, USA) HPLC system. The retention times were used for a qualitative analysis, and peak areas were used for a quantitative analysis.

### 2.5. ELISA Analysis of ROS Level and Enzyme Activities

ROS level, and the enzyme activities of β-glucosidase, UDP-glucuronosyltransferase, cytochrome P450s, superoxide dismutase, peroxidase and catalase in the grasshopper samples were measured following the method described by Li et al. [33]. In brief, grasshopper samples were homogenized in 1 mL phosphate buffered saline (PBS), and the resulting suspension subjected to ultrasonication to further break the cell membranes. After that, we centrifuged the homogenates for 15 min at 5000 rpm, collected the supernatants and stored at −20 °C until required for further analysis. The ELISA procedures were conducted according to the manufacturer’s instructions.

### 2.6. Field Cage Trial

A field cage study was conducted on an alfalfa area to evaluate the field survival of *C. abbreviatus* exposed to rutin and quercetin in June 2022. A total of 50 mesh cages (size: 1 m × 1 m × 1 m) were installed in this alfalfa area. All other plant species were removed to ensure that only *M. sativa* remained in these field cages. *M. sativa* plants were mainly at the vegetative stage, with heights ranging from ~53.2 to ~62.7 cm and dry biomass ranging from 132.4 to 167.8 g/m^2^. *M. sativa* in each cage was sufficient for grasshopper feeding until the end of the field experiment. Before adding *C. abbreviatus*, all potential natural enemies in these field cages were removed. A total of 1000 third instar nymphs of *C. abbreviatus* were collected and assigned randomly to 50 cages. Each cage included 20 healthy individuals. The prepared solutions of rutin and quercetin of different concentrations (0%, 0.001%, 0.01%, 0.1% and 1.0%) were applied evenly to grass *M. sativa* in field cages using a hand sprayer. In each cage, 100 mL of solution was sprayed on *M. sativa*. This field trial included ten treatments with five replications per treatment. The number of surviving individuals was determined on days 7 and 14 to calculate the survival rate (%) by the number of surviving individuals/the number of initial third instar individuals (n = 20).

### 2.7. Data Analyses

A one-way ANOVA was used to compare the difference in survival rate, body mass, development time, gene expression, ROS level, rutin and quercetin content, and enzyme activities in grasshoppers treated with the different concentrations of rutin or quercetin. A Student’s *t*-test was used to compare the differences in the grasshopper variables between rutin and quercetin treatments within the same concentration. All analyses were carried out using SAS version 9.0 (SAS Institute, Inc., Cary, NC, USA).

## 3. Results

### 3.1. Grasshopper Survival, Body Mass and Developmental Time 

**Figure 1:**
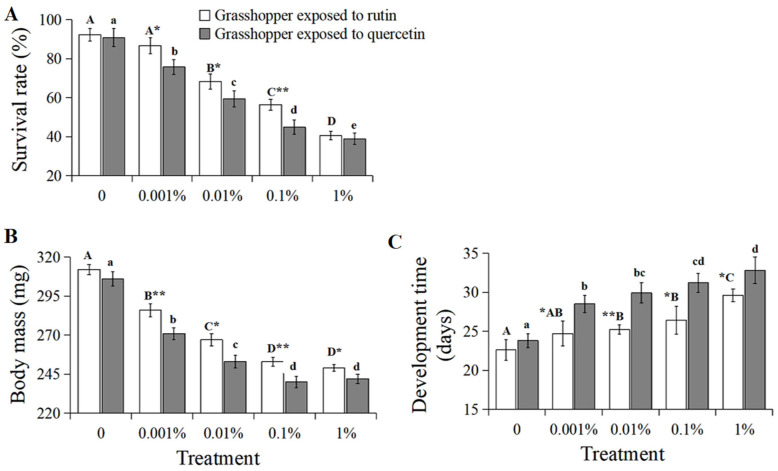
The survival (**A**), body mass (**B**) and developmental time (**C**) of *Calliptamus abbreviatus* exposed to rutin and quercetin. Bars with different uppercase letters indicate significant differences within the treatment with rutin, based on Turkey’s HSD analysis at *p* < 0.05. Bars with different lowercase letters indicate significant differences within the treatment with quercetin, based on Turkey’ s HSD analysis at *p* < 0.05. A Student’s *t*-test was used to compare the differences in grasshoppers treated with rutin and quercetin of the same concentration. * *p* < 0.05, ** *p* < 0.01.

The survival of *C. abbreviatus* negatively correlated with increased rutin and quercetin concentration, respectively (Figure 1A, grasshoppers exposed to rutin: R^2^ = 0.9816, *p* < 0.01; grasshoppers exposed to quercetin: R^2^ = 0.9794, *p* < 0.01). The survival rate of *C. abbreviatus* treated with 0–1% rutin significantly (*F* = 19.25, *df* = 4, 35, *p* < 0.05) decreased from 92.3% to 40.5%, with that of grasshoppers treated with 0–1% quercetin significantly (*F* = 20.68, *df* = 4, 35, *p* < 0.05) decreasing from 90.8% to 38.9%. In addition, grasshoppers treated with quercetin had a lower survival rate than rutin-treated grasshoppers at a concentration of 0.001–0.1%. 

The body mass of *C. abbreviatus* also inversely correlated with increased rutin and quercetin concentration (Figure 1B, grasshoppers exposed to rutin: R^2^ = 0.9359, *p* < 0.05; grasshoppers exposed to quercetin: R^2^ = 0.9082, *p* < 0.05). The body mass of grasshoppers treated with 0–1% rutin significantly (*F* = 18.32, *df* = 4, 35, *p* < 0.05) decreased from 312 mg to 249 mg, with that of grasshoppers treated with 0–1% quercetin decreasing from 306 mg to 242 mg. And grasshoppers treated with quercetin had a lower body mass than rutin-treated grasshoppers at a concentration of 0.001–1%. 

The developmental time of *C. abbreviatus* positively correlated with increased rutin and quercetin concentration (Figure 1C, grasshoppers exposed to rutin: R^2^ = 0.9280, *p* < 0.05; grasshoppers exposed to quercetin: R^2^ = 0.9099, *p* < 0.05). Developmental time significantly (*F* = 15.63, *df* = 4, 35, *p* < 0.05) increased from 22.6 days to 29.6 days for increased rutin concentration from 0% to 1%, with that of grasshoppers treated with 0–1% quercetin increasing from 23.8 days to 32.8 days (*F* = 19.31, *df* = 4, 35, *p* < 0.05). And grasshoppers treated with quercetin had a slower developmental time than rutin-treated grasshoppers at a concentration of 0.001–1%. 

### 3.2. Gene Expressions in Grasshopper

**Figure 2:**
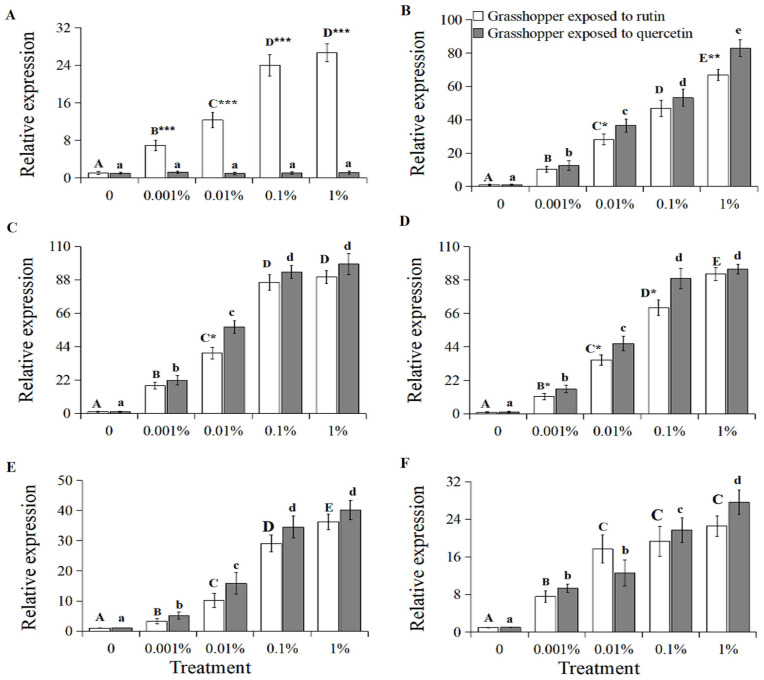
The relative gene expression of β-glucosidase (**A**), UDP-glucuronosyltransferase (**B**), cytochrome P450 6k1 (**C**), superoxide dismutase (**D**), peroxidase (**E**), and catalase (**F**) in *Calliptamus abbreviatus* exposed to rutin and quercetin. Bars with different uppercase letters indicate significant differences within rutin treatment, based on Turkey’s HSD analysis at *p* < 0.05. Bars with different lowercase letters indicate significant differences within quercetin treatment, based on Turkey’ s HSD analysis at *p* < 0.05. A Student’s *t*-test was used to compare the differences between grasshoppers treated with rutin and quercetin at the same concentration. * *p* < 0.05, ** *p* < 0.01, *** *p* < 0.001.

The expression of the grasshopper β-glucosidase gene positively correlated with increased rutin concentration (Figure 2A, grasshopper exposed to rutin: R^2^ = 0.9816, *p* < 0.01). The relative gene expression in 0–1% rutin-treated grasshoppers increased significantly to 26.63 (*F* = 18.27, *df* = 4, 35, *p* < 0.05). It was also higher in rutin-treated than in quercetin-treated grasshoppers at a concentration of 0.001–1%. 

The gene expressions of the grasshopper UDP-glucuronosyltransferase, cytochrome P450 6k1, superoxide dismutase, peroxidase and catalase also positively correlated with increased rutin and quercetin concentration (Figure 2B–F, R^2^ > 0.92, *p* < 0.05). The relative expressions of these genes in 0–1% rutin-treated grasshoppers increased significantly to 66.82, 89.8, 91.92, 36.25 and 22.53 (*F* = 8.56–26.57, *df* = 4, 35, *p* < 0.05), respectively. Likewise, the relative expressions of these genes in the 0–1% quercetin-treated grasshoppers increased significantly to 82.97, 98.3, 95.27, 40.18 and 27.61(*F* = 9.32–21.13, *df* = 4, 35, *p* < 0.05), respectively. Among them, the gene expressions of UDP-glucuronosyltransferase, cytochrome P450 6k1 and superoxide dismutase in grasshoppers treated with quercetin were higher than those in rutin-treated grasshoppers at a concentration of 0.001–1%. 

### 3.3. Grasshopper Rutin and Quercetin Content

**Figure 3:**
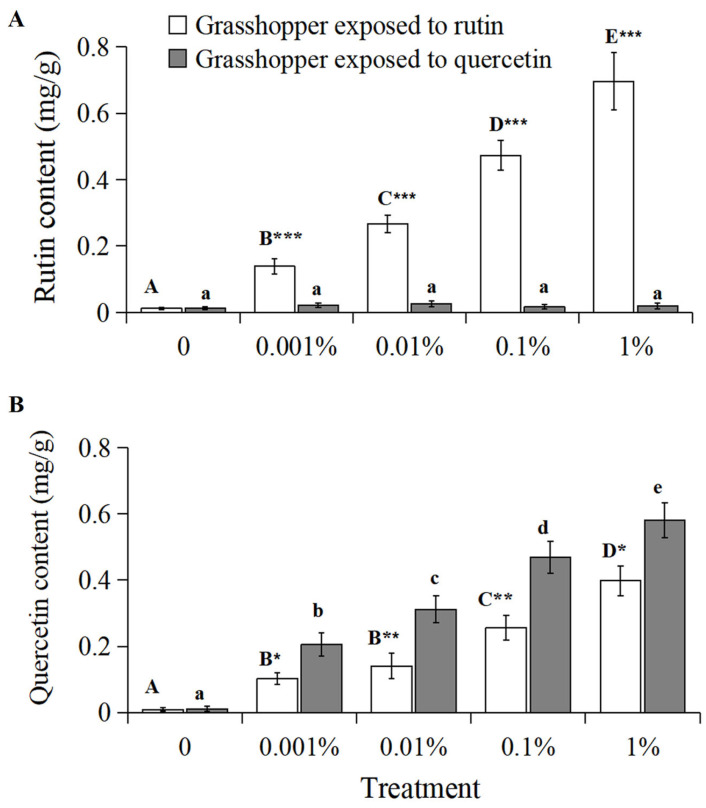
The rutin (**A**) and quercetin content (**B**) in *Calliptamus abbreviatus* exposed to rutin and quercetin. Bars with different uppercase letters indicate significant differences within rutin treatment, based on Turkey’s HSD analysis at *p* < 0.05. Bars with different lowercase letters indicate significant differences within quercetin treatment, based on Turkey’ s HSD analysis at *p* < 0.05. A Student’s *t*-test was used to compare the differences between grasshoppers treated with rutin and quercetin at the same concentration. * *p* < 0.05, ** *p* < 0.01, *** *p* < 0.001.

The rutin content in grasshoppers treated with rutin positively correlated with increased rutin concentration (Figure 3A, R^2^ = 0.9814, *p* < 0.01). The rutin content in grasshoppers treated with 0–1% rutin significantly (*F* = 17.93, *df* = 4, 35, *p* < 0.05) increased from 0.012 mg/g to 0.696 mg/g. And grasshoppers treated with rutin had a higher rutin content than quercetin-treated grasshoppers at a concentration of 0.001–1%. 

The quercetin content in grasshoppers treated with rutin and quercetin positively correlated with increased rutin and quercetin concentration, respectively (Figure 3B, grasshoppers exposed to rutin: R^2^ = 0.9633, *p* < 0.01; grasshoppers exposed to quercetin: R^2^ = 0.9906, *p* < 0.05). The quercetin content in grasshoppers treated with 0–1% rutin significantly (*F* = 13.18, *df* = 4, 35, *p* < 0.05) increased from 0.009 mg/g to 0.398 mg/g. The quercetin content in grasshoppers treated with 0–1% quercetin significantly (*F* = 14.45, *df* = 4, 35, *p* < 0.05) increased from 0.011 mg/g to 0.581 mg/g. Grasshoppers treated with quercetin had a higher quercetin content than rutin-treated grasshoppers at a concentration of 0.001–1%. 

### 3.4. Grasshopper ROS Level

**Figure 4:**
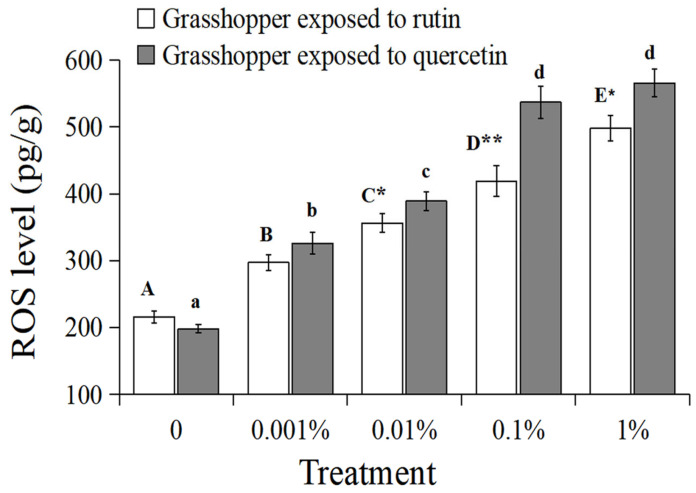
The ROS level in *Calliptamus abbreviatus* exposed to rutin and quercetin. Bars with different uppercase letters indicate significant differences within rutin treatment, based on Turkey’s HSD analysis at *p* < 0.05. Bars with different lowercase letters indicate significant differences within quercetin treatment, based on Turkey’ s HSD analysis at *p* < 0.05. A Student’s *t*-test was used to compare the differences between grasshoppers treated with rutin and quercetin at the same concentration. * *p* < 0.05, ** *p* < 0.01.

The ROS level in the grasshoppers had a significantly positive relationship with rutin and quercetin concentration (Figure 4, grasshoppers exposed to rutin: R^2^ = 0.9969, *p* < 0.01; grasshoppers exposed to quercetin: R^2^ = 0.9677, *p* < 0.01). The ROS level in grasshoppers treated with 0–1% rutin significantly increased (*F* = 16.56, *df* = 4, 35, *p* < 0.05) from ~216 pg/g to ~498 pg/g. The ROS level in grasshoppers treated with 0–1% quercetin significantly increased (*F* = 19.29, *df* = 4, 35, *p* < 0.05) from ~198 pg/g to ~566 pg/g. And the ROS level in grasshoppers treated with quercetin was higher than that in rutin-treated grasshoppers at a concentration of 0.01–1%. 

### 3.5. Grasshopper Enzyme Activity

**Figure 5:**
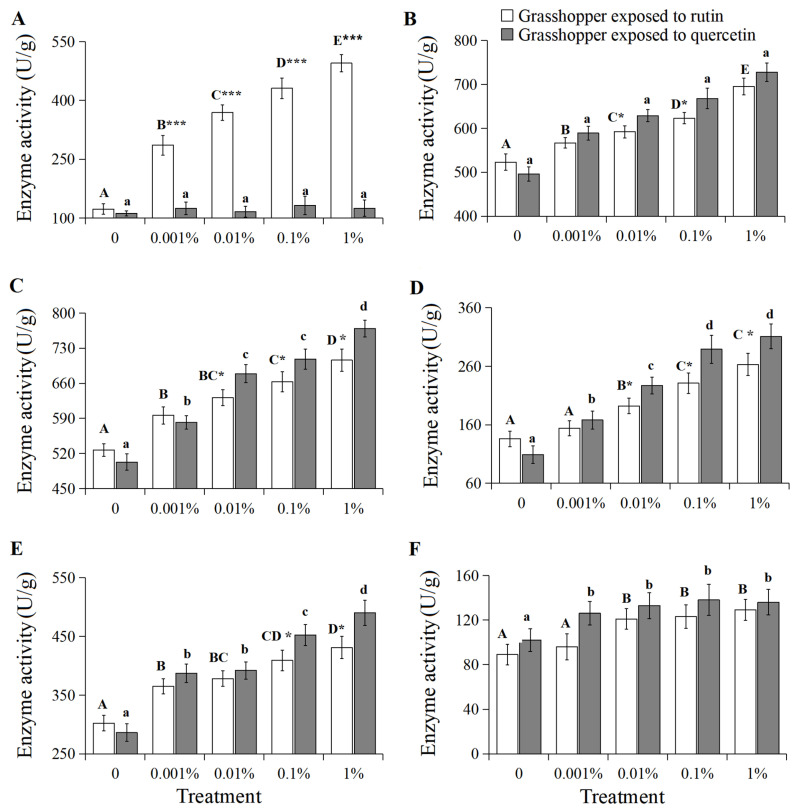
β-glucosidase (**A**), UDP-glucuronosyltransferase (**B**), cytochrome P450s (**C**), superoxide dismutase (**D**), peroxidase (**E**) and catalase (**F**) activities in *Calliptamus abbreviatus* exposed to rutin and quercetin. Bars with different uppercase letters indicate significant differences within rutin treatment, based on Turkey’s HSD analysis at *p* < 0.05. Bars with different lowercase letters indicate significant differences within quercetin treatment, based on Turkey’ s HSD analysis at *p* < 0.05. Student’s *t*-tests were used to compare the differences between grasshoppers treated with rutin and quercetin at the same concentration. * *p* < 0.05, *** *p* < 0.001.

β-glucosidase activity positively correlated with increased rutin concentration (Figure 5A, R^2^ = 0.9505, *p* < 0.01). β-glucosidase activity in 0–1% rutin-treated grasshoppers increased significantly from 123 U/g to 495 U/g (*F* = 18.92, *df* = 4, 35, *p* < 0.05). And grasshoppers treated with rutin had higher β-glucosidase activity than in quercetin-treated grasshoppers at a concentration of 0.001–1%. 

UDP-glucuronosyltransferase, cytochrome P450 6k1, superoxide dismutase, peroxidase and catalase activities also positively correlated with increased rutin and quercetin concentrations (Figure 5B–E, R^2^ > 0.9, *p* < 0.05). Their activities in 0–1% rutin-treated grasshoppers increased significantly from 523 U/g, 527 U/g, 136 U/g, 302 U/g and 89 U/g to 695 U/g, 706 U/g, 263 U/g, 431 U/g and 129 U/g, respectively (*F* = 7.86–23.69, *df* = 4, 35, *p* < 0.05). They also increased significantly from 496 U/g, 503 U/g, 109 U/g, 286 U/g and 102 U/g to 728 U/g, 769 U/g, 311 U/g, 490 U/g and 136 U/g, respectively (*F* = 9.21–19.63, *df* = 4, 35, *p* < 0.05) in 0–1% quercetin-treated grasshoppers. The enzyme activities in grasshoppers treated with quercetin were higher than those in rutin-treated grasshoppers at a concentration of 0.001–1%, with the exception of catalase. 

### 3.6. Grasshopper Survival Rate in Field Cage

**Figure 6:**
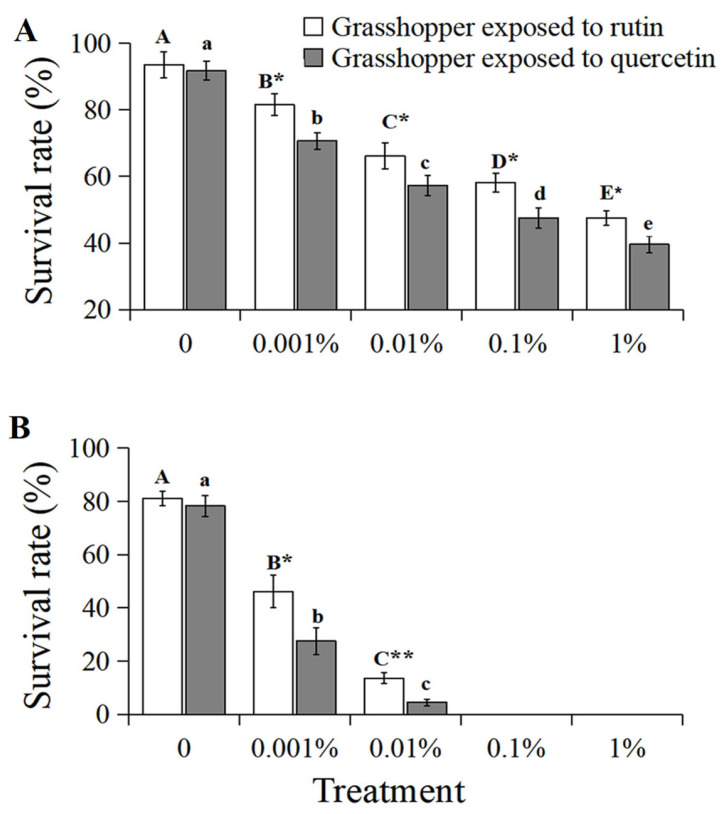
The survival rate of *Calliptamus abbreviatus* exposed to rutin and quercetin at 7 d (**A**) and 14 d (**B**) in field cages. Bars with different uppercase letters indicate significant differences within rutin treatment, based on Turkey’s HSD analysis at *p* < 0.05. Bars with different lowercase letters indicate significant differences within quercetin treatment, based on Turkey’ s HSD analysis at *p* < 0.05. A Student’s *t*-test was used to compare the differences between grasshoppers treated with rutin and quercetin at the same concentration. * *p* < 0.05, ** *p* < 0.01.

The survival rate of grasshoppers at 7 d negatively correlated with an increased concentration of rutin and quercetin (R^2^ > 0.9601, *p* < 0.05, Figure 6A). The survival rate of 0–1% rutin-treated grasshoppers at 7 d decreased significantly from 93.6% to 47.5% (*F* = 17.13, *df* = 24, *p* < 0.05). Likewise, that of quercetin-treated grasshoppers decreased significantly from 91.8% to 39.6% (*F* = 9.82, *df* = 24, *p* < 0.05). And grasshoppers treated with quercetin had a lower survival rate at 7 d than that of rutin-treated grasshoppers at a concentration of 0.001–1%.

The survival rate of 0–0.01% rutin-treated grasshoppers at 14 d decreased significantly from 81.2% to 13.6% (*F* = 15.01, *df* = 24, *p* < 0.05, Figure 6B). Also, that of quercetin-treated grasshoppers decreased significantly from 78.3% to 4.5% (*F* = 12.16, *df* = 24, *p* < 0.05, Figure 6B). Grasshoppers treated with quercetin had a lower survival rate at 14 d than that of rutin-treated grasshoppers at concentrations of 0.001% and 0.01%. No survival was recorded at 14 d for the 0.1% and 1% concentration treatments of these two chemicals. 

## 4. Discussion

In recent years, with the aggravation of grasshoppers in China, the discovery and utilization of plant-derived compounds for the sustainable control of these insects have attracted much attention. In this study, we found that rutin and quercetin significantly reduced the survival rate and inhibited the development of *Calliptamus abbreviatus*, indicating that they were toxic to *C. abbreviatus* and have the potential to be developed as biopesticides. In addition, we found that quercetin showed stronger toxicity than rutin to grasshoppers, as indicated by a lower survival rate and slower development than those of rutin.

Generally, herbivorous insects produce large amounts of detoxification enzymes, such as UDP-glucuronosyltransferase and cytochrome P450s, in response to toxic plant-derived compounds. This is to convert or degrade them to reduce tissue damage and enhance their survival under toxicity stress [23,25,26,27,34,35]. For example, cytochrome P450s and UDP-glucuronosyltransferase activities in *Spodoptera litura*, *Helicoverpa zea* and *Manduca sexta* treated with toxic plant-derived compounds were significantly higher than those in the control group [36,37,38,39,40]. In this study, we also found that the gene expressions and activities of these two detoxification enzymes increased significantly after treatment with rutin and quercetin, indicating that they played an important role in the resistance of *C. abbreviatus* to the toxic compounds. We also found that *C. abbreviatus* showed higher detoxification enzyme activities to convert or degrade toxic quercetin than for rutin. The detoxification of harmful plant compounds is a high-energy process [41,42,43,44,45,46]. Therefore, the detoxification of quercetin in grasshoppers may have been energetically costly, which resulted in lower survival and development than in those treated with rutin. However, the molecular mechanism underlying the detoxification of rutin and quercetin in *C. abbreviatus* requires further research.

Exposure to toxic compounds usually leads to an increase in ROS concentration in organisms, triggering oxidative damage and programmed apoptosis [47,48]. Therefore, ROS concentration can reflect the degree of environmental stress [7,21,22]. In this study, ROS levels in grasshoppers significantly increased after treatment with toxic rutin and quercetin, with higher levels recorded under quercetin treatment. This also supports the observation that quercetin showed stronger toxicity than rutin in *C. abbreviatus*. ROS levels in insects are mainly regulated by superoxide dismutase, peroxidase and catalase [24,25]. They are induced to synergically reduce ROS levels, to maintain the normal growth and development of organisms [21,23,49]. These protective enzymes also play an important role in the resistance to oxidative stress caused by toxic plant compounds. In this study, expressions of the genes and superoxide dismutase, peroxidase and catalase activities in grasshoppers all increased after treatment with rutin and quercetin; however, those in quercetin-treated grasshoppers were higher than those in rutin-treated grasshoppers.

Gene expression and related enzyme function were the underpinning mechanisms of herbivorous insects resisting toxic compounds [16]. Those changed genes were the basis of genetic adaptation and allowed the rapid induction of arrays of broader or more robustly active digestive, antioxidant or detoxifying enzymes in herbivorous insects after the consumption of toxic compounds [23]. These rapid biochemical responses to toxic compounds are vital for insect survival and growth. In this study, we also found that those gene expressions were in accordance with their related enzyme activities. *C. abbreviatus* treated by rutin or quercetin had high gene expression and related enzyme activity associated with digestion, oxidation resistance and detoxification, which implied that they play an important role in the resistance of *C. abbreviatus* to toxic rutin and quercetin in order to survive.

Rutin (C_27_H_30_O_16_) is an important flavonoid glycoside widely distributed in plants and is composed of quercetin and glucose ligands. It is the glycoside form of quercetin [29,30,31]. β-glucosidase, a hydrolase found in the membrane of animal midgut epithelial cells, plays a role in the metabolism of carbohydrates taken by animals, especially in the transmembrane metabolism of flavonoid glycosides. Mature β-glucosidase can hydrolyze flavonoid glycosides into free aglygen and glucose ligands [17,50]. In this study, we also found that *C. abbreviatus* showed increased gene expression and β-glucosidase activity when exposed to rutin, which corresponded with a higher quercetin content in grasshoppers. β-glucosidase maybe involved in the hydrolyzation of rutin to quercetin in *C. abbreviatus*. However, this requires further study. 

Rutin and quercetin reduced the survival and inhibited the development of *C. abbreviatus*. However, the higher toxicity of quercetin suggests its potential to be developed as a biopesticide for grasshopper control. In this study, we preliminarily evaluated the effects of rutin and quercetin on the physiological response of *C. abbreviatus*. We propose that further research should be conducted to understand the complex relationship between *C. abbreviatus* and these two plant-derived compounds, to accelerate their application in pest control. For example, more studies on the feeding behavior of grasshoppers should be conducted before the application of control measures based on quercetin and rutin in the field. Studies on the consumption of quercetin and rutin-treated plants by grasshoppers should also be conducted. The fact that the body mass of the grasshoppers dropped together with an increase in quercetin and rutin concentrations suggested that these botanicals may have an inhibited ability to feed. The next step should be to provide more toxicology tests to determine the lethal medium concentration (LC_50_), lethal medium dose (LD_50_) lethal medium time (LT_50_), and effective methods for applying rutin or quercetin to control *C. abbreviatus*.

## Data Availability

The data that support the findings of this study are available from the corresponding author upon reasonable request.

## Supplementary Materials

The following supporting information can be downloaded at: https://www.mdpi.com/article/10.3390/insects15020095/s1, Table S1: Designed sequences of gene primers for real time PCR.
