# Supplementary material for: The Survival and Physiological Response of Calliptamus abbreviatus Ikonn (Orthoptera: Acrididae) to Flavonoids Rutin and Quercetin
Source: Insects. 2024 Feb 1;15(2):95. doi: 10.3390/insects15020095 (PMC10888613; doi:10.3390/insects15020095)
Supplement: Supplementary file 1 [file insects-15-00095-s001.zip › insects-2836388-supplementary.pdf]

The survival and physiological response of *Calliptamus abbreviatus* Ikonn (Orthoptera: Acrididae) to flavonoid rutin and quercetin

Xunbing Huang[1], Li Zheng[1,2], Yuyue Wang[1],

[1] College of Agriculture and Forestry Science, Linyi University, Linyi 276000, P.R. China;

[2] Key Laboratory of Natural Enemies Insects, Ministry of Agriculture and Rural Affairs, Jinan 250100, China

Corresponding author, e-mail: xunbingh@163.com; zhengli64@126.com

Table S1 Designed sequences of gene primers for real time PCR

Gene description	Sequence of primers (5' to 3')	
β-glucosidase	Forward	TCGGAACTGGCTGCATCGATG
	Reverse	CCTCGTACCAGCAGCTGAGC
UDP-glucuronosyltransferase 2B	Forward	CGTGATAGGAAGCCTCGTT
	Reverse	TGTGGAAGTGGGAGAAGGA
cytochrome P450s 6k	Forward	ACAAGGAAGATGGCAGGA
	Reverse	GGGCTCTACTCAATACTCCG
superoxide dismutase	Forward	ATTCTCAGTCCAGACACGG
	Reverse	TCTCAGGGTAATGATGGTATCC
peroxidase	Forward	CCTGAGTGGTGTCTACGTGA
	Reverse	GCAGCGACGCAGCTCATCGG
catalase	Forward	GGACTAGGGACGTTAGGCTA
	Reverse	TAGACGTGAGGAGGGCGTGG
β-actin	Forward	CGTCATCTTCTCACGGTTG
	Reverse	TCTGGCACCACACCTTCTAC
